# Contaminant-Activated Visible Light Photocatalysis

**DOI:** 10.1038/s41598-018-19972-0

**Published:** 2018-01-30

**Authors:** Vijay Krishna, Wei Bai, Zhao Han, Akihiro Yano, Abhinav Thakur, Angelina Georgieva, Kristy Tolley, Joseph Navarro, Ben Koopman, Brij Moudgil

**Affiliations:** 1 Particle Engineering Research Center, University of Florida, Gainesville, FL 32611 USA; 2 Department of Materials Science and Engineering, University of Florida, Gainesville, FL 32611 USA; 3 Department of Environmental Engineering Sciences, University of Florida, Gainesville, FL 32611 USA; 4 Nagoya Institute of Technology, Tajimi, Gifu, 5070033 Japan; 5 Village on the Isle, Venice, FL 34285 USA; 6 NanoHygienix LLC, Sarasota, FL 34236 USA; 7 Present Address: Department of Biomedical Engineering, Lerner Research Institute, Cleveland Clinic, Cleveland, OH 44195 USA; 8 Present Address: Sinmat, Inc., Gainesville, FL 32653 USA; 9 Present Address: Western Digital Corporation, San Jose, CA 95138 USA; 10 Present Address: Mitsubishi Plastics, Inc., Tokyo, Japan; 11 Present Address: Freeport-McMoRan Copper & Gold Inc., Bagdad, AZ 86321 USA; 12 Present Address: Synergy Sales Co., Sarasota, FL 34232 USA

## Abstract

Pristine titanium dioxide (TiO_2_) absorbs ultraviolet light and reflects the entire visible spectrum. This optical response of TiO_2_ has found widespread application as white pigments in paper, paints, pharmaceuticals, foods and plastic industries; and as a UV absorber in cosmetics and photocatalysis. However, pristine TiO_2_ is considered to be inert under visible light for these applications. Here we show for the first time that a bacterial contaminant (*Staphylococcus aureus—*a MRSA surrogate) in contact with TiO_2_ activates its own photocatalytic degradation under visible light. The present study delineates the critical role of visible light absorption by contaminants and electronic interactions with anatase in photocatalytic degradation using two azo dyes (Mordant Orange and Procion Red) that are highly stable because of their aromaticity. An auxiliary light harvester, polyhydroxy fullerenes, was successfully used to accelerate photocatalytic degradation of contaminants. We designed a contaminant-activated, transparent, photocatalytic coating for common indoor surfaces and conducted a 12-month study that proved the efficacy of the coating in killing bacteria and holding bacterial concentrations generally below the benign threshold. Data collected in parallel with this study showed a substantial reduction in the incidence of infections.

## Introduction

Patients and visitors in healthcare facilities can acquire infections by direct or indirect contact with common surfaces (room door handles, bed rails, taps, sterile packaging, mops, ward fabrics and plastics, keyboards and telephones) that have become contaminated with pathogenic microbes^1^. Making these surfaces microbe-unfriendly can break the cycle of contamination and infection. Antimicrobial coatings that slowly release toxic silver or copper ions, currently in clinical trials^2–5^, have limited lifetime, are difficult to apply and are costly^6–9^. Further, copper surfaces were unable to reduce bacterial concentrations to the benign level in clinical trials^2,4^.

TiO_2_ photocatalysis has attracted intense interest for applications in self-cleaning and antimicrobial coatings as TiO_2_ can completely mineralize organic contaminants including microorganisms and the process produces no toxic by-products^10–12^. Further, TiO_2_ is environmentally benign and inexpensive^13,14^. Unfortunately, TiO_2_, which is an excellent photocatalyst under UV light, has very limited capability for visible light absorption^11,15,16^.

Extension of TiO_2_ photocatalysis to visible light is a highly active area of research^17–24^. Major approaches are: *1)* creation of defects within the TiO_2_ crystalline structure, such as oxygen or titanium vacancies or substitutions. Techniques include doping (with elements such as, carbon, nitrogen, sulfur or phosphorous), annealing in reducing atmospheres and synthesis in the presence of reductants^24–27^; *2)* creation of defects at the TiO_2_ surface. Techniques include surface hydrogenation, plasma treatment and surface amination^26,28,29^; *3)* combination of visible light harvesters with the TiO_2_. Techniques include co-synthesis with materials such as gold, copper and quantum dots^24,30,31^, and mixing with organic dyes, such as methylene blue, porphyrin and metal-quinoline complexes^24,32–34^. The three methods differ with respect to the site of visible light absorption and concomitant exciton generation: throughout the modified crystal, at the surface of the modified crystal or in the light harvester. With respect to the third method, exciton-exciton annihilation within light harvesters is suppressed by transfer of excited electrons to TiO_2_, which has high electron affinity^15^.

Among the techniques for extending the TiO_2_ photocatalysis in visible region, mixing with organic dyes is by far the simplest and is the basis for dye-sensitized solar cells^35^. Unfortunately, the dyes are photocatalytically degraded, leading to a short-term benefit^24^. In this report, we show that model organic contaminants (two organic dyes, Mordant Orange and Procion Red, and the bacterium, *Staphylococcus aureus*) in contact with pristine TiO_2_ (anatase) can harvest visible light and transfer electrons to TiO_2_, resulting in photocatalytic degradation of the contaminants. We also show, for the first time, that a natural, bacterial contaminant (*S. aureus*) with very low levels of light absorption is inactivated on pristine TiO_2_ by the same mechanism. In this mechanism, which we refer to as contaminant activated photocatalysis, the rate of photocatalytic degradation depends on the extent of visible light absorption. This new information is utilized to design transparent, contaminant activated photocatalytic coatings for prevention of healthcare-acquired infections.

## Results and Discussion

### Characterization of anatase TiO_2_

**Figure 1:**
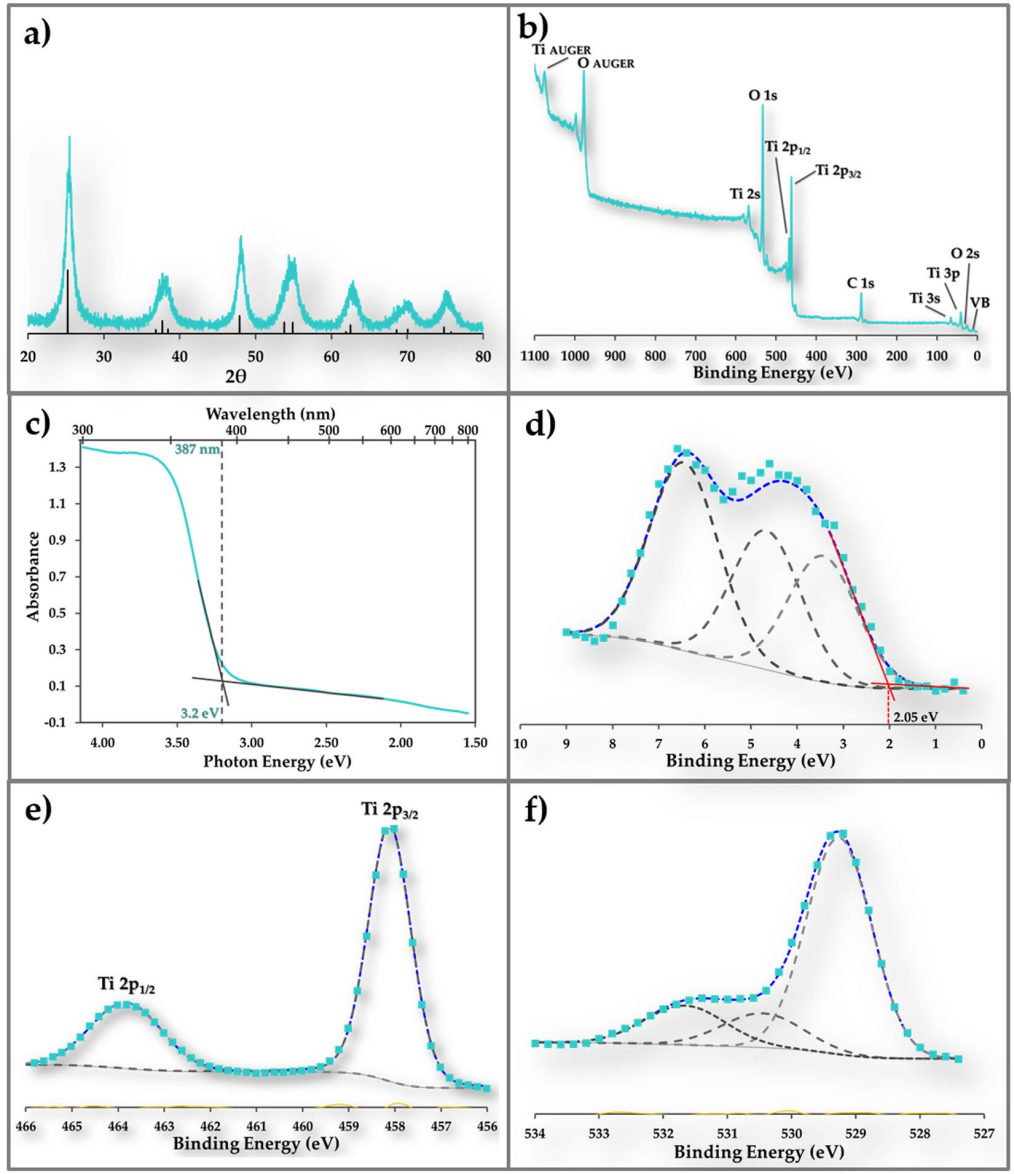
Physical characterization of pristine anatase. (**a**) XRD spectrum of anatase used in this study. The black lines are data for standard anatase. (**b**) XPS spectrum for anatase used in this study. Adventitious carbon was used as reference. (**c**) Ground state absorption spectrum of anatase showing bandgap (3.2 eV) estimation. (**d**) XPS high resolution valence band spectrum for anatase showing the edge of valence band at 2.05 eV. (**e**) XPS high resolution Ti 2p spectrum for anatase showing the Ti 2p_3/2_ peak at 458.1 eV and Ti 2p_1/2_ peak at 463.8 eV. (**f**) XPS high resolution O 1 s spectrum for anatase. Peaks at 529.3 eV, 530.5 eV and 531.7 eV are attributed to oxygen in the TiO_2_ lattice, surface Ti-OH and physisorbed water, respectively.

The X-ray diffraction pattern for TiO_2_ (Fig. 1a) has characteristic peaks at 25.4°, 37.8°, 48.0°, 53.7°, 54.9°, 62.8° and 75.1° that are assigned to (101), (004), (200), (105), (211), (204) and (215) crystal planes of anatase. The crystallite size of TiO_2_ was estimated from the Scherrer equation to be 7 nm. The X-ray photoelectron spectroscopic (XPS) analysis of TiO_2_ (Fig. 1b) indicated the presence of titanium, oxygen and adventitious carbon. The band gap was determined to be 3.2 eV from ground-state absorption spectrum (Fig. 1c) using UV-Vis spectrophotometer with integrating sphere and the valence band maximum energy was determined to be 2.05 eV from valence band spectrum (Fig. 1d) using XPS. These values are consistent with reported values^24,25,27,36^. The Ti 2p spectrum (Fig. 1e) exhibits the Ti 2p_3/2_ peak at 458.1 eV and Ti 2p_1/2_ peak at 463.8 eV, which are consistent with reported Ti^4+^ values^25,26^. No Ti^3+^ peaks were present. The O 1 s spectrum (Fig. 1f) has peaks at 529.3 eV, 530.5 eV and 531.7 eV that are attributed to oxygen in TiO_2_ lattice, surface Ti-OH and physisorbed water, respectively^26,37^. The binding energy differences between core emission lines and the valence band maxima Δ*E*_Ti-VB_ = 456.05 eV and Δ*E*_O-VB_ = 527.25 eV are consistent with the reported values for anatase^36^.

### Electronic interactions between contaminants and TiO_2_

**Figure 2:**
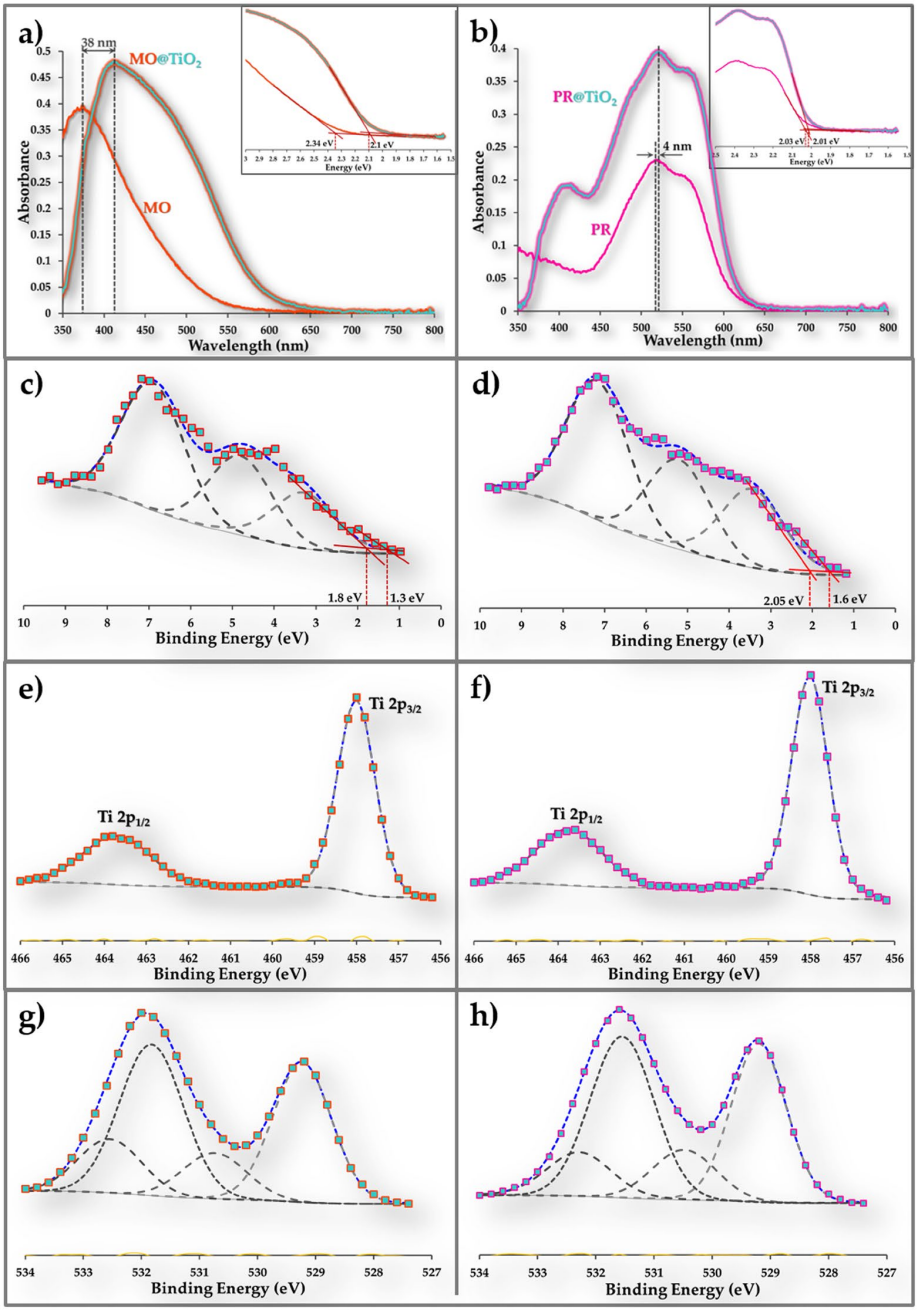
Electronic interactions of contaminant with anatase. (**a**) Ground state absorption spectra for Mordant Orange on anatase coating (MO@TiO_2_) and Mordant Orange on silica coating (MO). The inset shows bandgap (2.34 eV for MO and 2.1 eV for MO@TiO_2_) estimation. (**b**) Ground state absorption spectra for Procion Red on anatase coating (PR@TiO_2_) and Procion Red on silica coating (PR). The inset shows bandgap estimation (2.03 eV for PR and 2.01 eV for PR@TiO_2_). (**c**) XPS high resolution valence band spectrum for anatase in contact with MO shows valence band maximum at 1.8 eV with a tail at 1.3 eV. (**d**) XPS high resolution valence band spectrum for anatase in contact with PR shows valence band maximum at 2.05 eV with a tail at 1.6 eV. (**e**) XPS high resolution Ti 2p spectrum for anatase in contact with MO showing the Ti 2p_3/2_ peak at 458.1 eV and Ti 2p_1/2_ peak at 463.8 eV. (**f**) XPS high resolution Ti 2p spectrum for anatase in contact with PR showing the Ti 2p_3/2_ peak at 458.1 eV and Ti 2p_1/2_ peak at 463.8 eV. (**g**) XPS high resolution O 1 s spectrum for anatase in contact with MO. Peaks at 529.2 eV, 530.7 eV, 531.8 eV and 532.5 eV are attributed to oxygen in the TiO_2_ lattice, surface Ti-OH, physisorbed water and oxygen in MO, respectively. (**h**) XPS high resolution O 1 s spectrum for anatase in contact with PR. Peaks at 529.2 eV, 530.5 eV, 531.5 eV and 532.3 eV are attributed to oxygen in the TiO_2_ lattice, surface Ti-OH, physisorbed water and oxygen in PR, respectively.

To test our hypothesis that contaminant mediated absorption of visible light activates TiO_2_ photocatalysis, we chose two model contaminants that absorb visible light at different wavelengths—mordant orange with peak absorption at 374 nm (Fig. 2a) and procion red with peak absorption at 518 nm (Fig. 2b). Tiles were coated with TiO_2_ and the contaminants were pipetted onto the coated tiles, allowed to spread and dried in the dark (see supplementary information). Absorption spectra for the model contaminants in contact with TiO_2_ (MO@TiO_2_ and PR@TiO_2_) are shown in Fig. 2a,b, respectively. For comparison, the absorption spectra for model contaminants in contact with silica coatings (MO and PR) are also shown. Absorption peaks of the model contaminants on silica coating are the same as the peaks obtained for the contaminants in aqueous solution, suggesting that there was no interaction between either mordant orange or procion red and silica. Adsorption of contaminants onto the TiO_2_ coated surfaces cause bathochromic (red) shift and broadening of the light absorption peaks and shifting of the absorption edges to lower energies. These changes to light absorption suggest electron transfer from model organic contaminants to TiO_2_ and are consistent with observations reported for dye adsorption on TiO_2_^32^. The peak and absorption edge for mordant orange are red-shifted by 38 nm and 0.24 eV, respectively, and the peak and absorption edge for procion red are red-shifted by 4 nm and 0.02 eV, respectively. The greater red-shift for mordant orange suggests stronger electronic interaction with TiO_2_. As shown in Fig. 2c,d, the valence band maxima for TiO_2_ in contact with the contaminants exhibit both a shift to lower energies and addition of a tail, similar to observations for anatase synthesized with dopants or defects^25,27,28,38^. The valence band maximum for mordant orange + TiO_2_ is 1.8 eV with a tail at 1.3 eV, whereas the valence band maximum for procion red + TiO_2_ is 2.05 eV with a tail at 1.6 eV. The greater shift in valence band maximum for mordant orange + TiO_2_ is consistent with the greater bathochromic shift and suggests stronger electronic interactions. The Ti 2p spectra are not appreciably changed by addition of model contaminants to the TiO_2_ (Fig. 2e,f). However, a strong secondary peak is observed in the O 1 s spectra, corresponding to oxygen in the model contaminants (Fig. 2g,h). These results indicate that contacting model contaminant with pristine TiO_2_ effects changes in electronic properties of photocatalyst similar to those obtained by doping TiO_2_ with elements such as carbon or nitrogen^24^; co-synthesizing TiO_2_ with light harvesters such as phosphorous or quantum dots^25^; or surface modifying TiO_2_ by, for example, surface hydrogenation^28^.

### Confirmation of mechanism of contaminant activated photocatalysis

**Figure 3:**
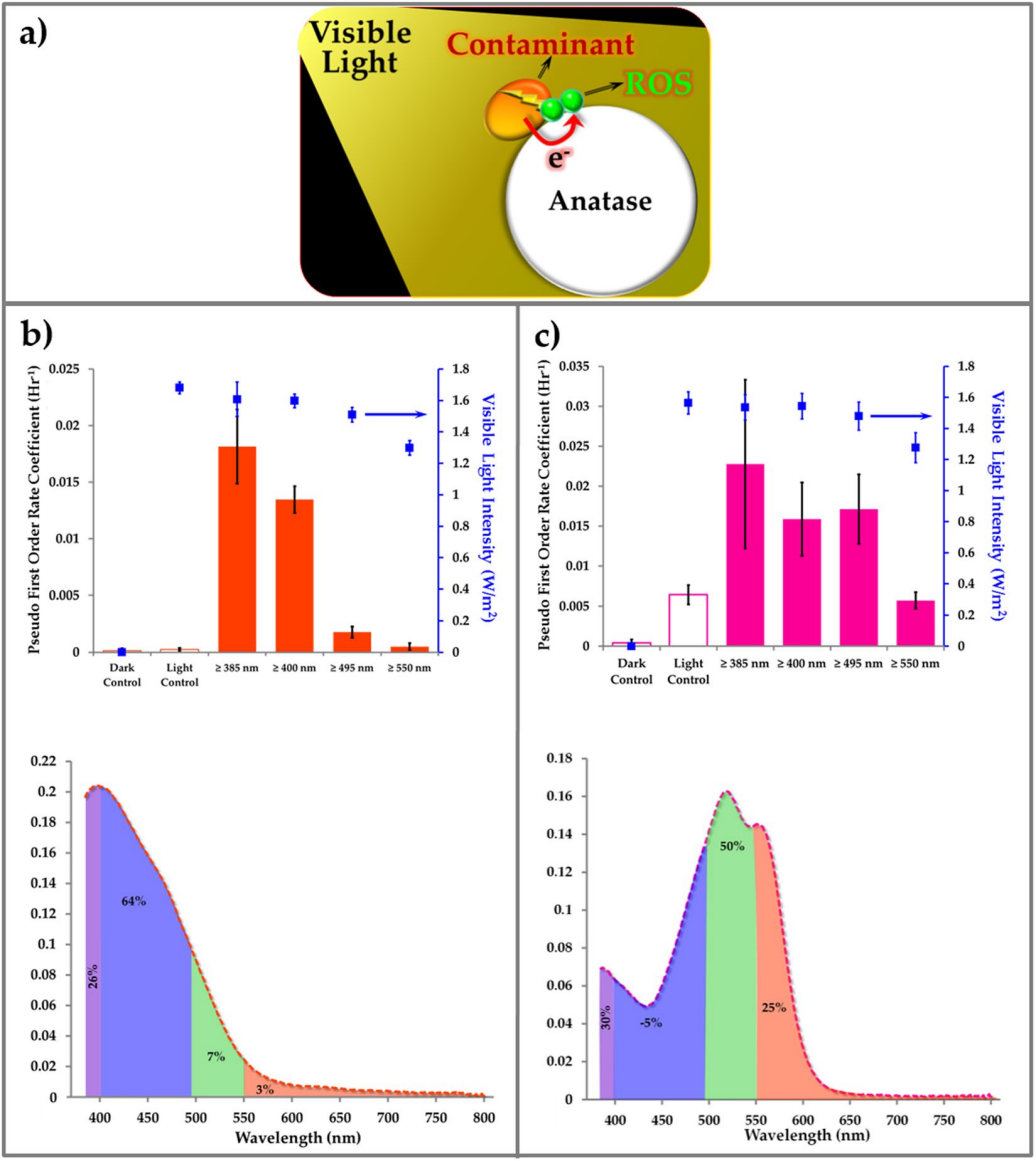
Contaminant-activated visible light photocatalysis. (**a**) Hypothetical mechanism of contaminant-activated visible light photocatalysis. (**b**) and (**c**) Effect of light wavelength on photocatalytic degradation rate of Mordant Orange (MO) and Procion Red (PR) on anatase coatings. Light control uses silica as an inert coating. Relative contribution of wavelength bands to the overall degradation rate (0.018 hr^−1^ for MO and 0.023 hr^−1^ for PR). The wavelength bands evaluated were determined by the lower bound of emission (385 nm) from the fluorescent lamps used for illumination and the cutoff wavelengths (400, 495, 550 nm) of the longpass optical filters employed. The ratio of contaminants MO and PR was 1 µg contaminant per 10 µg of TiO_2_ or SiO_2_. N = 4.

The hypothetical mechanism of contaminant activated visible light photocatalysis is shown in Fig. 3a. Visible light is absorbed by the contaminant, generating electron-hole pairs (excitons). Excited electrons are scavenged from the contaminant by anatase, which has high electron affinity^15^. These electrons react with oxygen and water at the surface of TiO_2_ particles forming highly labile oxygen species, such as superoxide radicals and hydroxyl radicals (ROS) that, in turn, decompose the contaminant. If this mechanism is correct, contaminant degradation should depend on overlap between the contaminant’s absorption spectrum and the incident light spectrum. To confirm this dependency, we used optical longpass filters to, in effect, create several wavelength bands in the region between 385 nm (the lower limit of emission of the fluorescent lamps employed experimentally) and 800 nm (nominally the upper limit of visible light). Experiments were carried out under room light produced by fluorescent lamps, which emit light with wavelength greater than 385 nm (see Figure S1 for spectral distribution of the lamp). According to our hypothesis, mordant orange, which absorbs most strongly at 400 nm, should be degraded fastest with light in this wavelength region. This was confirmed experimentally (Fig. 3b). Relative contribution of wavelength bands to the overall degradation rate are shown in bottom graph of Fig. 3b. The wavelength bands evaluated were determined by the lower bound of emission (385 nm) from the fluorescent lamps used for illumination and the cutoff wavelengths (400, 495, 550 nm) of the longpass optical filters employed. The wavelength band of 400 to 495 nm contributed to 64% of overall degradation rate (0.018 hr^−1^). Similarly, procion red, with an absorption peak at 520 nm, was degraded most rapidly within the wavelength band of 495 to 550 nm (Fig. 3c). Controls consisting of dye on a photocatalytically inert surface (silica) and dye on TiO_2_ in darkness showed effectively no activity, indicating that both TiO_2_ and visible light are required for photocatalytic degradation. The optical filters introduced a slight decrease in overall light intensity with increasing cutoff wavelength, but a modest decrease on this same order was shown to have negligible effect on photocatalytic degradation rate (Figure S2). Thus, it is seen that the rate of photocatalysis under visible light correlates strongly with light absorption by organic contaminants.

**Figure 4:**
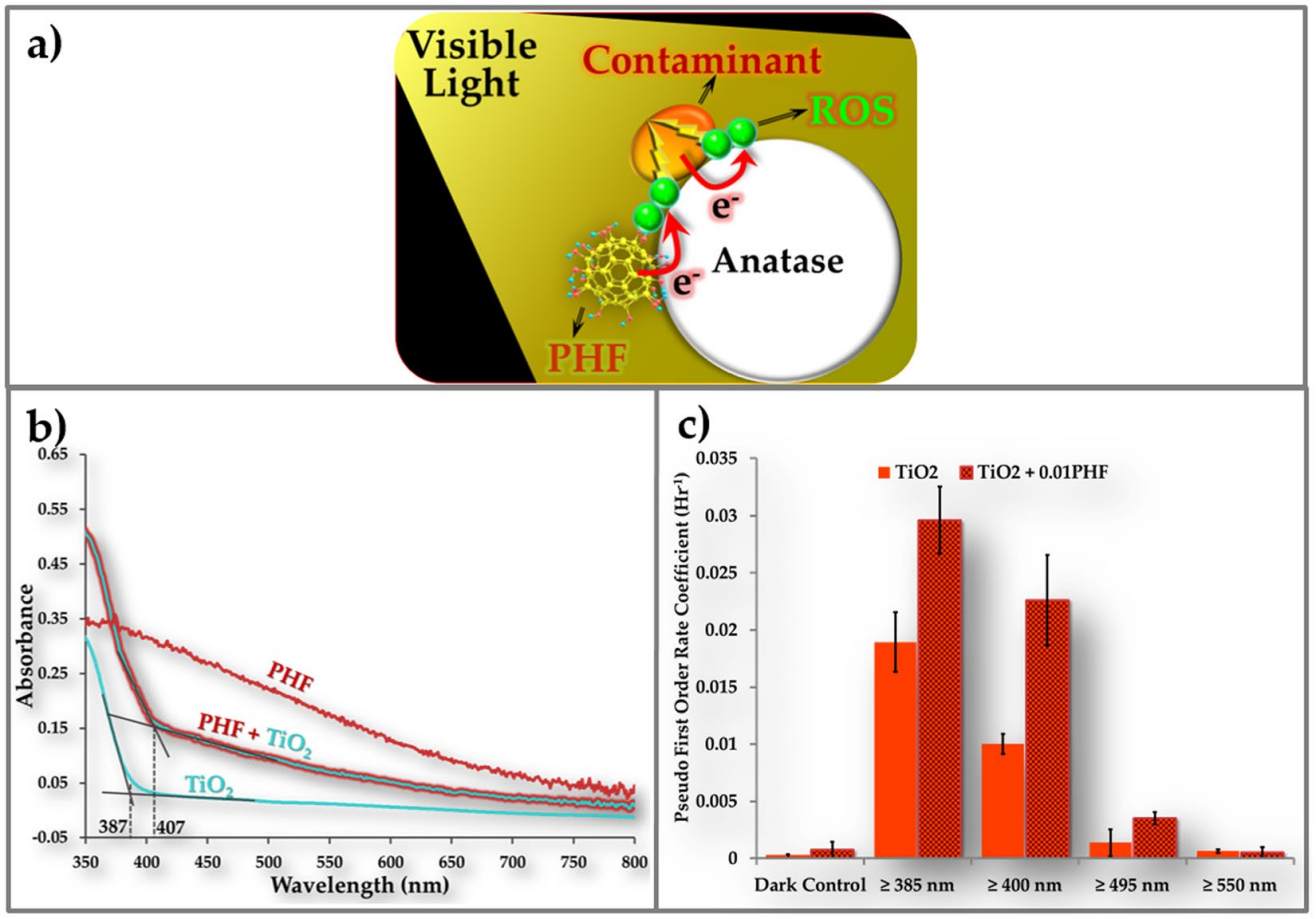
Role of auxiliary light harvester in contaminant-activated photocatalysis. (**a**) Hypothetical mechanism of contaminant-activated visible light photocatalysis with auxiliary light harvester polyhydroxy fullerene. (**b**) Ground state absorption spectra for anatase (TiO_2_), polyhydroxy fullerenes (PHF) on silica and anatase coating (PHF + TiO_2_). (**c**) Pseudo first order rate coefficients for degradation of Mordant Orange dye on anatase (TiO_2_) and anatase + 0.01 (w/w) PHF (TiO_2_ + 0.01PHF) coatings. Dark control measures the ability of the photocatalytic coatings to degrade dye in the dark. N = 10.

An auxiliary visible light harvester should be able to enhance photocatalytic degradation through increased exciton generation and, concomitantly, electron transport to TiO_2_ where ROS are formed (Fig. 4a). Polyhydroxy fullerenes (PHF), because of its broadband absorption (Fig. 4b) and high degree of stability^39,40^, should be an excellent candidate for this role. As shown in Fig. 4c, incorporation of PHF in the TiO_2_ coating increased the contaminant degradation rate by up to 2.5 times, depending on wavelength. The largest effect was observed with light between 400 nm and 495 nm. Cutoff beyond 550 nm led to very low rates with or without PHF.

### Coating optimization

**Figure 5:**
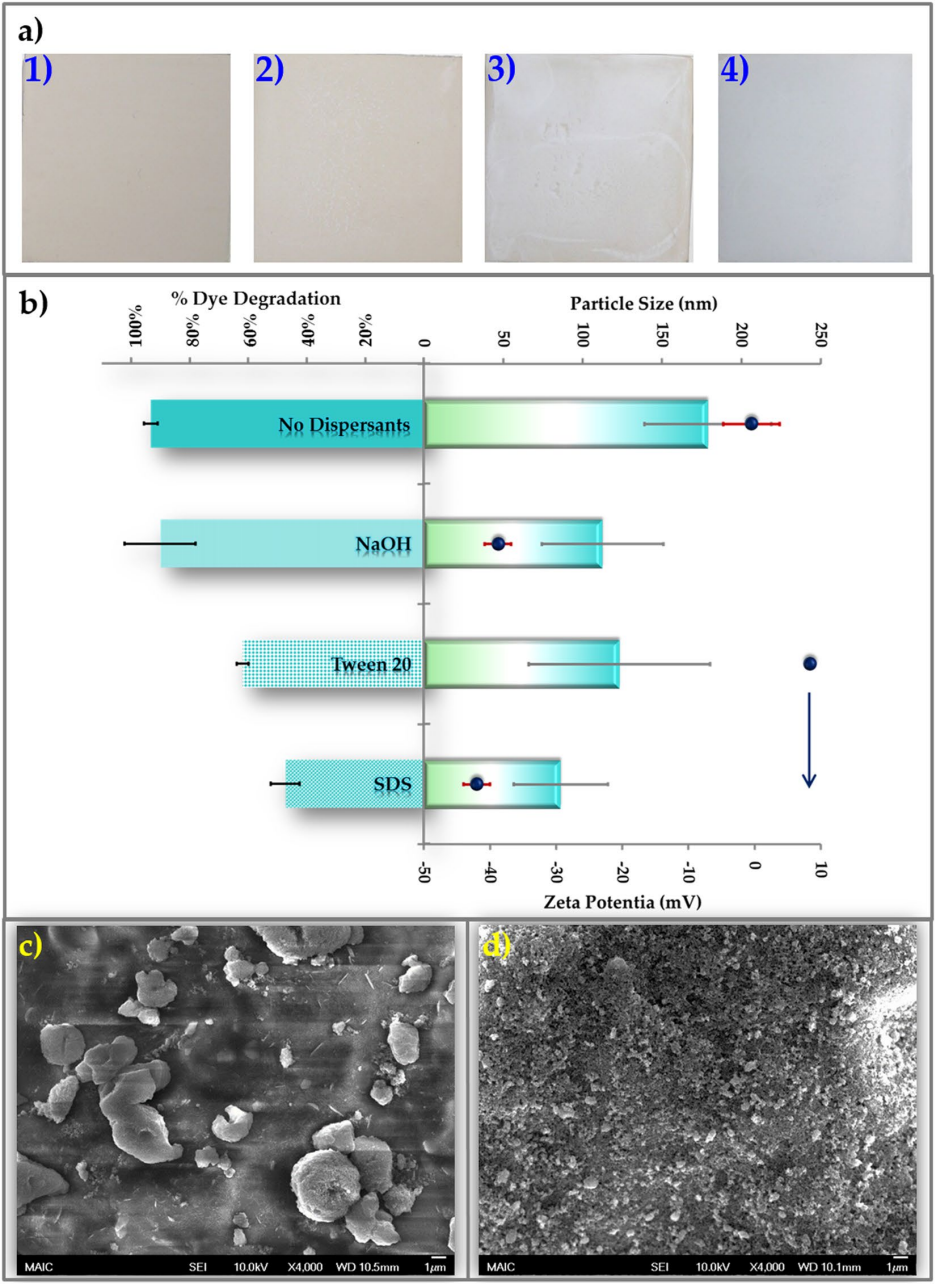
Design of transparent coating. (**a**) Changes in appearance of tile surfaces achieved with application of TiO_2_ coating at particle loadings of **1)** 0 mg/cm^2^; **2)** 0.128 mg/cm^2^; **3)** 1.28 mg/cm^2^; and **4)** 6.4 mg/cm^2^. (**b**) Effect of different dispersants on particle size and zeta potential of TiO_2_ formulation and performance of contaminant-activated photocatalysis with Procion Red. (**c**) and (**d**) Scanning electron micrographs of TiO_2_ coatings prepared from formulations **(c)** without any dispersants and **(d)** stabilized with 0.01 M NaOH as dispersant.

Coating formulation was optimized to obtain a transparent and uniform photocatalytic coating as well as a stable formulation. Transparent photocatalytic coatings were obtained with a particle loading equal to 128 µg/cm^2^, giving a nominal thickness of 0.25 µm (Fig. 5a)^10^. Surfactants were investigated to disperse the formulation, but were found to inhibit contaminant activated photocatalysis (Fig. 5b). Electrostatic dispersion with NaOH (pH 9–10) provided a stable formulation (Figure S3) without compromising photocatalytic activity (Fig. 5b). In the absence of dispersant, the coating consisted of 10–100 µm agglomerates (Fig. 5c), whereas the agglomerate size in the electrostatically dispersed coating was less than 1 µm (Fig. 5d).

A commercial rutile phase of TiO_2_ with mean crystallite size of 22 nm was investigated as a bottom coating because of its observed tendency to form dense, uniform layer on ceramic tile. A dual phase coating prepared by applying a layer of rutile followed by a layer of anatase was compared to a two-layer coating consisting solely of anatase. The dual phase (rutile/anatase) coating performed slightly better than anatase (anatase/anatase) coating and was therefore adopted for the bacterial inactivation experiments (Figure S4).

### Activation of visible light photocatalysis by MRSA surrogate

**Figure 6:**
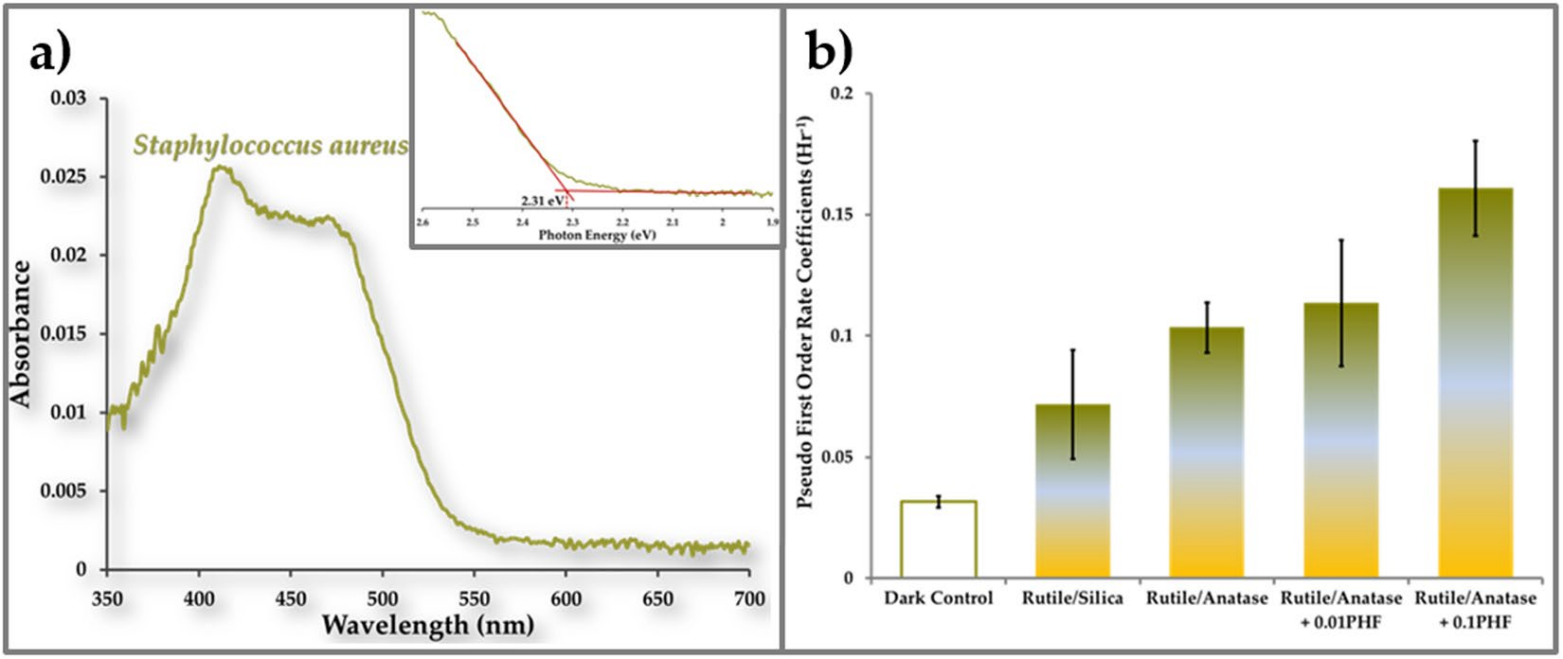
Bacteria-activated photocatalysis. (**a**) Ground state absorption spectrum for *Staphylococcus aureus* calculated from reflectance of *S. aureus* deposited on a tile surface. The inset shows bandgap estimation. (**b**) Pseudo first order rate coefficients for inactivation of *Staphylococcus aureus* on various coatings. Dark control measures the ability of the rutile/anatase + 0.1 (w/w) PHF coatings to inactivate bacteria in the dark. Light control measures the ability of rutile/silica coatings to inactivate bacteria in light. The ratio of bacteria was ~80 CFU per 1 µg of TiO_2_. N = 6.

A benign strain of the bacterium, *Staphylococcus aureus* (ATCC 25923), was chosen as a surrogate for MRSA. As shown in Fig. 6a, *S. aureus* is a very weak absorber of blue light (absorption peak at 411 nm; 0.0256 absorbance at 10^8^ CFU/cm^2^; absorption edge at 2.31 eV). Thus, it provided a severe test of the ability of a microbial contaminant to activate TiO_2_ photocatalysis under visible light.

Dual phase (rutile bottom layer/ anatase top layer) TiO_2_ coating was prepared on tiles and 0.1 mL of *S. aureus* suspension (2–3 × 10^5^ CFU/mL) was pipetted onto each coated tile surface and allowed to spread, giving a surface loading of ~8000 CFU/cm^2^. One set of tiles with photocatalytically inert silica substituted for the anatase top layer was also prepared. The tiles were dried in the dark in a biosafety cabinet for 3 hours (see supplementary methods).

As shown in Fig. 6b, control (dark) experiments with *S. aureus* on TiO_2_ coated tiles had inactivation rate of 0.031 ± 0.002 hr^−1^, which is attributable to desiccation. The rutile/silica coating under visible light inactivated *S. aureus* at a rate of 0.072 ± 0.02 hr^−1^, which is significantly greater (α = 0.001) than the dark control. Since rutile absorbs visible light up to a wavelength of 409 nm, the observed inactivation could be independent of light harvesting by *S. aureus*. However, in the rutile/anatase coating, the *S. aureus* inactivation rate was significantly (α = 0.001) increased to 0.103 ± 0.01 hr^−1^, providing confirmation of contaminant activated photocatalysis. Addition of auxiliary light harvester (PHF) increased the bacterial inactivation rate by up to 1.55 times (0.161 ± 0.01 hr^−1^ for rutile/anatase + 0.1 PHF).

Photocatalysis inactivates cells by degrading the cell surface. The *S. aureus* used in the present study expresses a capsule, which protects the cell-surface. Most MRSA strains do not express a capsule^41^, and therefore should be more susceptible to contaminant activated photocatalysis.

### Testing of optimized coating in a beta facility

**Figure 7:**
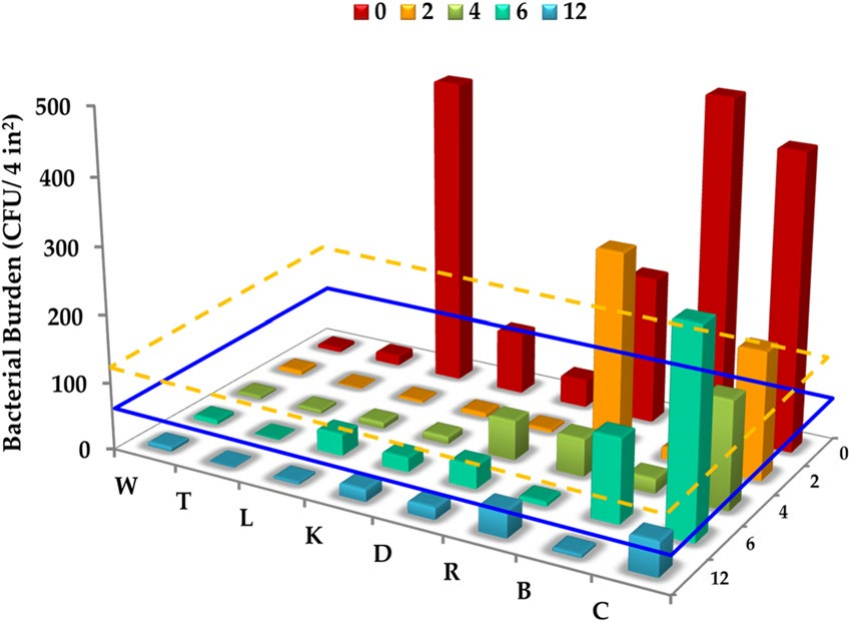
Beta facility testing. Reduction in bacterial burden on surfaces with antimicrobial coating. For a given surface, the bars represent counts (n = 3) at times from 0 to 12 months. W = Wall; T = Thermostat; L = Locker; K = Knob; D = Soap Dispenser; R = Bathroom Rail; B = Bed Rail; C = Counter. The blue dashed line indicates the threshold of microbial counts for benign surfaces, and the yellow dashed line indicates the average microbial counts on copper surfaces in a clinical trial^2^.

The optimized antimicrobial coating was further evaluated for its ability to control the microbial burden on surfaces in a beta facility. A commercial primer (BioShield NuTiO) replaced the bottom rutile layer in the optimized coating to provide a binding agent for the top antimicrobial layer. Surfaces (walls, counters, door knobs, lockers, thermostat, bed rail, bathroom rail and soap dispensers) were initially steamed to ensure primer adhesion, allowed to dry for 15 minutes, coated with primer, and allowed to dry for another 15 minutes. The top antimicrobial layer was then applied using commercial foggers. Selected surfaces were tested for bacterial count a total of five times: initially 1 hour after application of the antimicrobial coating, and subsequently 2, 4, 6 and 12 months after initial application of the coating. The bacterial counts over this 12-month period for each surface are shown in Fig. 7. The different surfaces are arranged along the x-axis of the figure, whereas the temporal variation of bacterial counts on each surface is indicated by a sequence of five bars, one for each time of sampling. The wall (W) and thermostat (T) had initial bacterial counts below the benign limit of 2.5 CFU/cm^2^ (64 CFU/ 4 sq.in.) proposed by Griffith *et al*.^2,42^. No significant change in bacterial count over the total 12 month period was observed on the wall, whereas the count on the thermostat decreased by 93% within 2 months. Initial bacterial counts on both lockers (L) and door knobs (K) greatly exceeded the benign limit. Counts on these surfaces decreased by 99% within 2 months and remained within the benign range for rest of the study. Soap dispensers (D) had low counts (within benign limit), possibly due to antimicrobial agents used in the soap. Initial bacterial counts on bathroom rails (R) and bed rails (B) were above the benign limit, with bed rails exhibiting the highest initial counts among all surfaces studied. Bacterial counts on the bathroom rails and bed rails decreased significantly throughout the study. Bacterial levels on the kitchen counter (C) were very high initially and remained above the benign limit for all but the last sampling. Of the five surfaces with initial bacterial counts above the benign level, only the kitchen counter failed to exhibit a consistent reduction in bacterial level. This could be due to frequent wiping (several times daily), which could have removed the antimicrobial coating. The incidence of acquired infections in the beta facility during the first six months of the study was reduced by 55% compared to the previous year. The beta facility results suggest that contaminant-activated photocatalysis can transform common indoor surfaces into antimicrobial surfaces with potential to break the cycle of contamination and infection.

## Conclusions

We have demonstrated for the first time that bacteria can sensitize pristine TiO_2_ to visible light, resulting in photocatalytic inactivation of the bacteria. Specific conclusions are as follow:

- Contacting pristine anatase with a visible-light-absorbing model contaminant creates, in effect, a photocatalyst with smaller bandgap and lower valence band maximum than the anatase.
- The degradation rate of model contaminants in contact with pristine anatase is strongly correlated with visible light absorption by the contaminants.
- *Staphylococcus aureus* with very weak visible light absorption was successfully inactivated on transparent, pristine TiO_2_ coatings.
- Bacteria that do not express a capsule, as well as viruses, should be at least as susceptible to contaminant activated photocatalysis as the test strain of *S. aureus*.
- Incorporation of an auxiliary light harvester (polyhydroxy fullerenes) accelerated degradation of model contaminants by up to 2.5 times and *S. aureus* by up to 1.5 times.
- Transparent photocatalytic coatings were obtained with a particle loading of 128 µg/cm^2^ (0.25 µm thickness).
- Electrostatic dispersion with NaOH (pH 9–10) provides a stable formulation without compromising photocatalytic activity.
- Application of photocatalytic coating to indoor surfaces decreased bacterial counts consistently to benign levels on five of six surfaces monitored over a period of 12 months. (Bacterial counts on two other surfaces were in the benign range at the outset of the study.)

Whether organic degradation is desired or undesirable, the potential of pristine TiO_2_ (particularly the anatase polymorph) to photocatalytically decompose organic materials under visible light must be taken into account in design of paints, pharmaceuticals, food additives, polymer composites, catalysts and antimicrobial coatings.

## Methods

Photocatalytic coating formulation was prepared by adding 10 mg of anatase to 10 mL of dilute NaOH (pH = 9.5) in a 20 mL scintillation vial wrapped with aluminum foil to prevent exposure to visible light. The suspension was sonicated for 30 minutes total. Rutile coating formulation and silica coating formulations were prepared in the same fashion. To prepare the anatase + PHF coating formulation, 10 mg of anatase was added to 9 mL of dilute NaOH (pH = 9.5) in a 20 mL scintillation vial wrapped with aluminum foil and the suspension was sonicated for 30 minutes. This suspension was then amended with 1 mL of solution containing either 0.1 or 1 mg of PHF and mixed with a magnetic stirrer for 10 minutes in dark. The two formulation with PHF are referred to as TiO_2_ + 0.01 PHF and TiO_2_ + 0.1 PHF, respectively. Coating formulations were applied to tiles within 1 hour of the preparation.

Ceramic tiles were utilized to evaluate the photocatalytic degradation of organic dye and inactivation of microbes. A volume of 0.4 mL of selected coating formulation was pipetted on the tile surface as the first coat. The coated surfaces were dried for one hour at 40 °C in dark. A second coat of same or different coating formulation was applied following the same procedure as described above. A total surface loading of 128 μg/cm^2^ was achieved with this procedure. Organic dye or *S. aureus* suspension was applied to the test surfaces. In case of organic dye, 0.02 mL of PR solution (2000 mg/L) or MO solution (2000 mg/L) was pipetted onto coated tiles and allowed to spread. The ratio of contaminants MO and PR was 1 µg contaminant per 10 µg of TiO_2_ or SiO_2_. The dye-coated tiles were dried at 50 °C for 20 minutes in dark before starting the performance evaluation. In case of *S. aureus*, 0.1 mL of *S. aureus* suspension (2–3 × 10^5^ CFU/mL) was pipetted onto each coated tile surface and allowed to spread, giving a surface loading of 6400–9600 CFU/cm^2^. The ratio of bacteria was ~80 CFU per 1 µg of TiO_2_. The tiles with *S. aureus* were dried in the dark in a biosafety cabinet for 3 hours.

The photocatalytic experiments were carried out under fluorescent lamps at visible light irradiance of 1.8–2.0 W/m^2^. The UVA irradiance (0.000 W/m^2^) was below the detection limit of the instrument consistent with no UV emission from the fluorescent lamp spectra. The temporal changes in dye concentration were determined by measuring the absorbance after predetermined times of exposure to visible light. The inactivation of *S. aureus* was evaluated by determining the viable counts after exposure to visible light.

The significance of visible light absorption by model contaminants was delineated by employing filters to limit the wavelengths of visible light available for absorption. Four 400 nm longpass filters (2″ × 2″) were joined to form a square of 4″ × 4″ held together by transparent tape. The coated tiles with PR or MO dyes were prepared as mentioned previously. In each experiment, four tiles were placed in a Petri dish. In case of neutral filter experiments, the Petri dishes were covered with their lids. In case of longpass filter experiments, The 4″ × 4″ filters were placed on the Petri dish (without lids) and the experiments were carried out as explained in previous section.

## Electronic supplementary material

Supplementary Methods and Figures

## References

[1] Page K, Wilson M, Parkin IP (2009). Antimicrobial surfaces and their potential in reducing the role of the inanimate environment in the incidence of hospital-acquired infections. Journal of Materials Chemistry.

[2] Schmidt MG (2012). Sustained reduction of microbial burden on common hospital surfaces through introduction of copper. Journal of clinical microbiology.

[3] Perez E (2014). Microbial biofilms on needleless connectors for central venous catheters: comparison of standard and silver-coated devices collected from patients in an acute care hospital. Journal of clinical microbiology.

[4] Salgado CD (2013). Copper surfaces reduce the rate of healthcare-acquired infections in the intensive care unit. Infection control and hospital epidemiology.

[5] Lederer JW, Jarvis WR, Thomas L, Ritter J (2014). Multicenter cohort study to assess the impact of a silver-alloy and hydrogel-coated urinary catheter on symptomatic catheter-associated urinary tract infections. Journal of wound, ostomy, and continence nursing: official publication of The Wound, Ostomy and Continence Nurses Society / WOCN.

[6] Dupont CL, Grass G, Rensing C (2011). Copper toxicity and the origin of bacterial resistance–new insights and applications. Metallomics.

[7] Knetsch MLW, Koole LH (2011). New Strategies in the Development of AntimicrobialCoatings: The Example of Increasing Usage of Silver and Silver Nanoparticles. Polymers-Basel.

[8] Novikov A (2012). Impact of catheter antimicrobial coating on species-specific risk of catheter colonization: a meta-analysis. Antimicrob Resist Infect Control.

[9] Rupp ME (2004). Effect of silver-coated urinary catheters: efficacy, cost-effectiveness, and antimicrobial resistance. Am J Infect Control.

[10] Bai W, Krishna V, Wang J, Moudgil B, Koopman B (2012). Enhancement of nano titanium dioxide photocatalysis in transparent coatings by polyhydroxy fullerene. Applied Catalysis B-Environmental.

[11] Fujishima, A., Hashimoto, K. & Watanabe, T. *TiO*_*2*_*Photocatalysis: Fundamentals and Applications*, (BKC Inc., Tokyo, 1999).

[12] Jacoby WA, Maness PC, Wolfrum EJ, Blake DM, Fennell JA (1998). Mineralization of bacterial cell mass on a photocatalytic surface in air. Environ Sci Technol.

[13] Robichaud CO, Uyar AE, Darby MR, Zucker LG, Wiesner MR (2009). Estimates of upper bounds and trends in nano-TiO_2_ production as a basis for exposure assessment. Environ Sci Technol.

[14] Weir A, Westerhoff P, Fabricius L, Hristovski K, von Goetz N (2012). Titanium Dioxide Nanoparticles in Food and Personal Care Products. Environ Sci Technol.

[15] Luttrell, T. *et al*. Why is anatase a better photocatalyst than rutile? - Model studies on epitaxial TiO_2_ films. *Sci Rep-Uk* **4**(2014). 10.1038/srep04043 PMC3918909 24509651

[16] Hashimoto K, Irie H, Fujishima A (2005). TiO2 photocatalysis: A historical overview and future prospects. Jpn J Appl Phys 1.

[17] Asahi R, Morikawa T, Ohwaki T, Aoki K, Taga Y (2001). Visible-light photocatalysis in nitrogen-doped titanium oxides. Science.

[18] Mrowetz M, Balcerski W, Colussi AJ, Hoffman MR (2004). Oxidative power of nitrogen-doped TiO2 photocatalysts under visible illumination. J Phys Chem B.

[19] Pelaez M (2012). A review on the visible light active titanium dioxide photocatalysts for environmental applications. Applied Catalysis B-Environmental.

[20] Yu JC (2005). Efficient visible-light-induced photocatalytic disinfection on sulfur-doped nanocrystalline titania. Environ Sci Technol.

[21] Low JX, Cheng B, Yu JG (2017). Surface modification and enhanced photocatalytic CO2 reduction performance of TiO2: a review. Appl Surf Sci.

[22] Wang SC (2017). Recent Progress on Visible Light Responsive Heterojunctions for Photocatalytic Applications. J Mater Sci Technol.

[23] Ansari SA, Khan MM, Ansaric MO, Cho MH (2016). Nitrogen-doped titanium dioxide (N-doped TiO2) for visible light photocatalysis. New J Chem.

[24] Fagan R, McCormack DE, Dionysiou DD, Pillai SC (2016). A review of solar and visible light active TiO2 photocatalysis for treating bacteria, cyanotoxins and contaminants of emerging concern. Mat Sci Semicon Proc.

[25] Ansari SA, Cho MH (2016). Highly Visible Light Responsive, Narrow Band gap TiO2 Nanoparticles Modified by Elemental Red Phosphorus for Photocatalysis and Photoelectrochemical Applications. Sci Rep.

[26] Bharti, B., Kumar, S., Lee, H. N. & Kumar, R. Formation of oxygen vacancies and Ti3 + state in TiO2 thin film and enhanced optical properties by air plasma treatment. *Sci Rep-Uk* **6**(2016). 10.1038/srep32355 PMC5004114 27572095

[27] Wang SB (2015). Titanium-Defected Undoped Anatase TiO2 with p-Type Conductivity, Room-Temperature Ferromagnetism, and Remarkable Photocatalytic Performance. J Am Chem Soc.

[28] Chen XB, Liu L, Yu PY, Mao SS (2011). Increasing Solar Absorption for Photocatalysis with Black Hydrogenated Titanium Dioxide Nanocrystals. Science.

[29] Burda C (2003). Enhanced nitrogen doping in TiO2 nanoparticles. Nano Letters.

[30] Li GS, Zhang DQ, Yu JC (2009). A New Visible-Light Photocatalyst: CdS Quantum Dots Embedded Mesoporous TiO2. Environ Sci Technol.

[31] Zheng ZK (2011). Facile *in situ* synthesis of visible-light plasmonic photocatalysts M@TiO2 (M = Au, Pt, Ag) and evaluation of their photocatalytic oxidation of benzene to phenol. Journal of Materials Chemistry.

[32] Hagberg DP (2007). Tuning the HOMO and LUMO energy levels of organic chromophores for dye sensitized solar cells. J Org Chem.

[33] Huang YW (2016). Effects of electronic structure and interfacial interaction between metal-quinoline complexes and TiO2 on visible light photocatalytic activity of TiO2. Applied Catalysis B-Environmental.

[34] Ismail AA, Bahnemann DW (2010). Metal-Free Porphyrin-Sensitized Mesoporous Titania Films For Visible-Light Indoor Air Oxidation. Chemsuschem.

[35] Hardin BE, Snaith HJ, McGehee MD (2012). The renaissance of dye-sensitized solar cells. Nat Photonics.

[36] Pfeifer V (2013). Energy Band Alignment between Anatase and Rutile TiO2. J Phys Chem Lett.

[37] Li, L. D. *et al*. Sub-10 nm rutile titanium dioxide nanoparticles for efficient visible-light-driven photocatalytic hydrogen production. *Nat Commun* **6**(2015). 10.1038/ncomms6881 25562287

[38] Feng, N. D. *et al*. Unravelling the Efficient Photocatalytic Activity of Boron-induced Ti3 + Species in the Surface Layer of TiO2. *Sci Rep-Uk* **6**(2016). 10.1038/srep34765 PMC5052528 27708430

[39] Krishna V, Noguchi N, Koopman B, Moudgil B (2006). Enhancement of titanium dioxide photocatalysis by water-soluble fullerenes. Journal of Colloid and Interface Science.

[40] Krishna V (2008). Mechanism of enhanced photocatalysis with polyhydroxy fullerenes. Applied Catalysis B-Environmental.

[41] Sutter DE (2011). Capsular serotype of Staphylococcus aureus in the era of community-acquired MRSA. FEMS immunology and medical microbiology.

[42] Griffith CJ, Cooper RA, Gilmore J, Davies C, Lewis M (2000). An evaluation of hospital cleaning regimes and standards. J Hosp Infect.

