# Supplementary material for: Contaminant-Activated Visible Light Photocatalysis
Source: Sci Rep. 2018 Jan 30;8:1894. doi: 10.1038/s41598-018-19972-0 (PMC5789862; doi:10.1038/s41598-018-19972-0)
Supplement: Supplementary file 1 — Supplementary Methods and Figures [file 41598_2018_19972_MOESM1_ESM.pdf]

# Contaminant-Activated Visible Light Photocatalysis

***Vijay Krishna[1, 2†], Wei Bai[1, 3†], Zhao Han[1, 2†], Akihiro Yano[1, 4], Abhinav Thakur[1, 2†], Angelina Georgieva[1], Kristy Tolley[5], Joseph Navarro[6†], Ben Koopman[1, 3] and Brij Moudgil[1, 2]***

*[1]Particle Engineering Research Center, [2]Department of Materials Science and Engineering, [3]Department of Environmental Engineering Sciences, University of Florida, Gainesville, FL 32611; [4]Nagoya Institute of Technology, Tajimi, Gifu 5070033, Japan; [5]Village on the Isle, Venice, FL 34285; [6]NanoHygienix LLC, Sarasota, FL 12345*

**†Present Address**

***Vijay Krishna:*** Department of Biomedical Engineering, Lerner Research Institute, Cleveland Clinic, Cleveland, OH 44195

***Wei Bai:*** Sinmat, Inc., Gainesville, FL 32653

***Zhao Han:*** Western Digital Corporation, San Jose, CA 95138

***Akihiro Yano:*** Mitsubishi Plastics, Inc., Tokyo, Japan

***Abhinav Thakur:*** Freeport-McMoRan Copper & Gold Inc., Bagdad, AZ 86321

***Joseph Navarro:*** Synergy Sales Co., Sarasota, FL 34232

**Corresponding Author**

Correspondence to Vijay Krishna (krishnv2@ccf.org)

## Supplementary Methods

### Materials and Methods

Titanium dioxide: 7 nm anatase and 22 nm rutile; and 15 nm silica were purchased from MKnano (Willamsville, NY). Mordant Orange (MO) and Procion Red MX-5B (PR) dyes were obtained from Sigma-Aldrich Inc. (St. Louis, MO). Ceramic tiles (1″ × 1″) were obtained from American Olean Inc. (Dallas, TX). Fullerene ($C_{60}$; 99% purity) was purchased from MER Corp. (Tuscon, AZ). A 50% NaOH solution was obtained from Sigma-Aldrich Inc. (St. Louis, MO). Polyhydroxy fullerene (PHF) was synthesized in laboratory according to the protocol of Georgeiva et al. [1]. Bioshield primer was obtained from Bioshield, Inc. (Fort Lauderdale, FL). Longpass filters (2″×2″) were obtained from Thorlabs Inc. (Newton, NJ). All other chemicals were purchased from Fisher Scientific (Pittsburg, PA).

### *Characterization of $TiO_2$ and contaminants*

The ground state absorption of $TiO_2$ and contaminants was determined with a Perkin-Elmer Lambda 1050 UV/Vis/NIR spectrophotometer with 150 mm integrating sphere. The band gap energy was determined from $E=hc/\lambda$, where $\lambda$ is the wavelength at which a strong cutoff in absorption is observed, h is the Plank constant ($4.14\times10^{-15}$ eV.s) and c is the speed of light in vacuum ($3.00\times10^{8}$ m/s). A white Spectralon® plate was used as the internal reference.

The crystal type and the size of crystallite was determined with X-ray diffraction on a APD 3720 diffractometer (Philips, Andover, MA) with Cu-K$\alpha$ radiation (40 kV, 25 mA) and diffracted beam monochromator, using a step scan mode with the step of 0.075° (2θ) and 4 s per step. Crystal structure was identified according to the database of International Centre for Diffraction Data (ICDD). The crystallite size was determined from the Scherrer equation:

$$L = \frac{K\lambda}{(B-b)\cos\theta}$$

Where L is the average crystallite size, K is the shape factor (0.9), $\lambda$ is the X-ray wavelength of Cu-$K_\alpha$ radiation (1.54 Å), B is the overall line broadening in radians at the full width at half maximum (FWHM) intensity, b is the line broadening in radians at the FWHM intensity caused by the instrument itself (0.07) and θ is the Bragg angle, i.e., the angle at which highest intensity was observed. X-ray diffraction pattern for anatase (Figure 1a) is in good agreement with the anatase crystalline structures given in the ICDD database.

The purity of $TiO_2$ powder was determined with X-ray photoelectron spectroscopy (XPS) (Perkin-Elmer 5100ESCA system). The data obtained was analyzed with AugerScan software (Thermo Fisher Scientific, Waltham, MA). X-ray photoelectron spectra indicated the presence of titanium, oxygen and adventitious carbon (Figure 1b). The valence band maximum and high resolution scan of $TiO_2$ with and without

contaminants were obtained with X-ray photoelectron spectroscopy (PHI Versaprobe 5000 system). The data was analyzed with MultiPak Data Reduction software (Physical Elestronics, Inc., Chanhassen, MN).

Specific surface area of anatase was measured under nitrogen using a NOVA 1200 with multipoint BET (Quantachrome Instruments, Boynton Beach, FL). $TiO_2$ powder was degassed and dried under vacuum at 110°C prior to measurement.

Scanning electron microscopy (JOEL 6335F FEG-SEM) was used to observe the ultrastructure of $TiO_2$ coating at the conditions of 10 kV accelerating voltage and 10 mm working distance.

***Coating formulation***

Photocatalytic coating formulation was prepared by adding 10 mg of anatase to 10 mL of dilute NaOH (pH = 9.5) in a 20 mL scintillation vial wrapped with aluminum foil to prevent exposure to visible light. The suspension was sonicated (Misonix Sonicator 3000, Farmingdale, NY) at the highest power level providing 180–200 W for 30 minutes total (10 min on/2 min off × 3). Rutile coating formulation and silica coating formulations were prepared in the same fashion. In case of anatase+PHF, 10 mg of anatase was added to 9 mL of dilute NaOH (pH = 9.5) in a 20 mL scintillation vial

wrapped with aluminum foil. The anatase suspension was sonicated as before followed by addition of 1 mL of PHF solution (1000 or 100 mg/L). The nanocomposites suspension was mixed with a magnetic stirrer for 10 minutes in dark. The two formulation with PHF are referred to as $TiO_2$ + 0.01 PHF and $TiO_2$ + 0.1 PHF, respectively. Coating formulations were applied to tiles within 1 hour of the preparation.

***Coating application***

Ceramic tiles were utilized to evaluate the photocatalytic degradation of organic dye and inactivation of microbes. Ceramic tiles (2.5 cm × 2.5 cm) were almond-colored with a matte finish. A volume of 0.4 mL of selected coating formulation was pipetted on the tile surface as the first coat. The coated surfaces were dried for one hour at 40°C in dark. A second layer of same or different coating formulation was applied following the same procedure as described above. A total surface loading of 128 $\mu g/cm^2$ was achieved with this procedure. The thickness of the coating was calculated according to Bai *et al.* [2] using the equation below

$$d = \frac{cV}{\rho A f_d}$$

Where $d$ = calculated thickness of $TiO_2$ coating, $c$ = aqueous concentration of $TiO_2$, $V$ = volume of $TiO_2$ suspension applied to the surface, $\rho$ = density of $TiO_2$, $A$ = area of surface and $f_d$ = maximum volume fraction of randomly close packed spheres (0.634).

***Bacteria Culture***

*Staphylococcus aureus* (ATCC 25923) was obtained from American Type Culture Collection (Manassas, VA). Tryptic soy agar and tryptic soy broth (Becton, Disckinson and Company, Sparks, MD) were used for culturing and enumerating the bacteria. A mass of 40 g Tryptic soy agar powder was suspended in 1 L of deionized water and mixed thoroughly with heating to the boiling point. The solution was then autoclaved at 120°C and 16 bar for 15 minutes. Plates were made by pouring the autoclaved agar into 100×15 mm sterile plastic Petri dishes (Fisher Scientific) and air dried in a laminar flow hood (LABCONCO purifier class 2 safe cabinet) for 24 hours. The dried agar plates were used immediately or stored in inverted position in a refrigerator at 4°C. Broth was prepared by adding a mass of 32 g tryptic soy broth powder to 1 L of deionized water and mixing thoroughly with heating to the boiling point. The broth was then autoclaved at 120°C and 16 bar for 20 minutes. Autoclaved broth was used immediately or stored in a refrigerator at 4°C. Phosphate-buffered saline (PBS) solution was prepared by dissolving 12.36 g $Na_2HPO_4$, 1.8 g $NaH_2PO_4$ and 85 g NaCl in 1000 mL of deionized water and then diluting 10× immediately before use. PBS/SDS solution was prepared by adding 0.576 g sodium dodecyl sulfate (SDS) to 1000 mL of PBS and then autoclaving at 120°C and 16 bar for 15 minutes.

The *S. aureus* culture was maintained by streaking the bacteria on tryptic soy agar in a Petri dish. The inoculated plate was inverted and incubated at 37°C for 24 hours. An inoculation loop was used to transfer a loop full of S. aureus from the plate to a 250 mL Erlenmeyer flask containing 100 mL of autoclaved tryptic soy broth. The flask was then placed in an incubator at 37°C for 24 hours. After 24 hours, a volume of 1 mL of bacterial suspension was added to ten centrifuge tubes each containing 1 mL of 50% glycerol as cryoprotectant. The mixture was then stored at -84°C until further use.

*S. aureus* was cultured by adding a 2 ml aliquot of *S. aureus* that was previously frozen at -84°C in 25% glycerol to a 250 mL Erlenmeyer flask containing 100 mL of sterile tryptic soy broth. The culture was incubated in a shaker-incubator at 150 rpm and 37°C for 24 hours. The suspension was washed three times with sterile deionized water and the final pellet was resuspended in 15 mL of deionized water.

The number of colony forming units (CFU) in a suspension of *S. aureus* was determined by serial dilution and viable plate counts. A series of 10-fold dilutions ($10^{-1}$ to $10^{-7}$) were prepared from the *S. aureus* suspension by adding 0.333 mL of sample to 3.0 mL sterile deionized water in a dilution tube, followed by vortexing for 10 seconds. A volume of 0.1 mL of diluted sample was spread over the surface of tryptic soy agar using a Teflon rod in each of three 100×15mm Petri dishes. The inoculated plates were inverted and

then incubated at 37°C for 24 hours. Where possible, results were taken from plates that contained between 30 and 300 colonies.

***Contaminant application***

Organic dye or *S. aureus* suspension was applied to the test surfaces. In case of organic dye, 0.02 mL of PR solution (2000 mg/L) or MO solution (2000 mg/L) was pipetted onto coated tiles and allowed to spread. The dye-coated tiles were dried at 50°C for 20 minutes in dark before starting the performance evaluation. In case of *S. aureus*, 0.1 mL of *S. aureus* suspension (2–3×$10^5$ CFU/mL) was pipetted onto each coated tile surface and allowed to spread, giving a surface loading of 6400–9600 CFU/$cm^2$. The tiles with *S. aureus* were dried in the dark in a biosafety cabinet for 3 hours.

***Coating performance evaluation***

The photocatalytic experiments were carried out under fluorescent lamps (General Electric model T8 Ultramax F28T8-SPX41) at visible light irradiance of 1.8–2.0 W/$m^2$. The spectral distribution of the fluorescent lamp is provided in Figure S1. The visible light and UVA irradiances were measured with a PMA 2140 Global detector or a PMA 2110 UVA detector attached to a PMA2110 meter (Solar Light Co., Glenside, PA). The UVA irradiance (0.000 mW/$cm^2$) was below the detection limit of the instrument consistent with no UV emission from the fluorescent lamp spectra.

A Perkin-Elmer Lambda 800 UV/VIS spectrophotometer with PELA-1000 reflectance accessory was used to measure light absorption in the range of 300 to 700 nm. The temporal changes in dye concentration were determined by measuring the absorbance (converted from measure reflectance) at 538 nm after predetermined times of exposure to visible light. Reflectance of coated or uncoated tile surfaces was measured with the Perkin-Elmer Lambda 800 with PELA-1000 Reflectance Spectroscopy Accessory (Perkin Elmer; Waltham, MA). Absorbance was calculated as the negative $\log_{10}$ of reflectance expressed as fraction. Coated tiles without dye were used as the internal reference in the measurement. Dye degradation was calculated according to:

$$\%\, Dye\ degradation = \frac{A_0 - A_t}{A_0} \times 100$$

Where $A_0$ is the calculated absorbance of dye on coated tile before exposure to visible light and $A_t$ is the absorbance of dye on coated tile after exposure to visible light at a given time.

The inactivation of *S. aureus* was evaluated by determining the viable counts after exposure to visible light. Bacteria were recovered by immersing a tile in 20 mL PBS/SDS solution within a polypropylene centrifuge tube and vortexing for 15 seconds. The tube was then sonicated at highest power for 1 minute. During sonication, the tube was immersed in a flowing water bath at 28°C. After sonication, the tube was vortexed for

15 seconds. The viable bacteria in a volume of 0.1 mL suspension from the centrifuge tube were enumerated as described previously. The inactivation was calculated with the following equation

$$\%\,Inactivation = \frac{CFU_0 - CFU_t}{CFU_0} \times 100$$

Where $CFU_0$ is the number of colonies at time zero and $CFU_t$ is the number of colonies after time t.

The % dye degradation and % inactivation data were modeled with first-order fits using following equation

$$\frac{C_0 - C_t}{C_0} = 1 - e^{-kt}$$

where $C_0$ and $C_t$ represent absorbance or colony forming units at times t=0 and t=t, respectively, and k is the first order rate coefficient, which was determined by least squares linear regression analysis.

***Photocatalysis with filters***

The significance of visible light absorption by contaminant was delineated by employing filters to limit the wavelengths of visible light available for absorption. Four 400 nm longpass filters (2″×2″) were joined to form a square of 4″×4″ held together by

transparent tape. The coated tiles with PR or MO dyes were prepared as mentioned previously. In each set of experiment, four tiles were placed in a Petri dish. In case of neutral filter experiments, the Petri dishes were covered with their lids. In case of longpass filter experiments, The 4″×4″ filters were placed on the Petri dish (without lids) and the experiments were carried out as explained in previous section.

**Coating in beta facility**

***Coating preparation***

The Bioshield primer was applied directly from the manufacturer's container. The $TiO_2$+0.1PHF nanocomposites was prepared by adding 200 mg of anatase to 180 mL of dilute NaOH solution (pH = 9-9.5). The $TiO_2$ suspension was sonicated (Misonix Sonicator 3000; Farmingdale, NY) at the highest power level providing 180-200 W for 30 minutes total (10 min on/ 2 min off ×3). A volume of 20 mL of PHF solution (1 mg/mL) was then added to $TiO_2$ suspension and mixed with magnetic stirrer for 10 minutes. This procedure was repeated to accumulate a total volume of 5 L.

***Coating application***

All surfaces (walls, ceilings, furniture, attached fixtures, etc.) were steamed prior to coating to remove contaminants and ensure adhesion of the coating. After 15 minutes of drying, Bioshield primer was applied using an electric fogger (Model 2600,

American Air & Water®, Inc.; Hilton Head Island, SC). After 15 minutes of drying time, the $TiO_2$+0.1PHF formulation was applied to all surfaces.

### *Microbial sampling procedure*

Sterile cotton swabs were used to collect microbes from selected surfaces. A swab was immersed in sterile deionized water, followed by wiping on selected surfaces (2 × 2 in) back and forward 5 times. Microbes adhered to the wetted cotton were streaked on Tryptic soy agar plates. The plates were inverted and then placed in a 37°C incubator. Colony forming units (CFU) were counted after 48 hours of incubation.

## References


1. Georgieva, A.T., *et al*. Polyhydroxy fullerenes. *Journal of Nanoparticle Research* **15**(2013).
2. Bai, W., Krishna, V., Wang, J., Moudgil, B. & Koopman, B. Enhancement of nano titanium dioxide photocatalysis in transparent coatings by polyhydroxy fullerene. *Applied Catalysis B-Environmental* **125**, 128-135 (2012).

# Supplementary Data

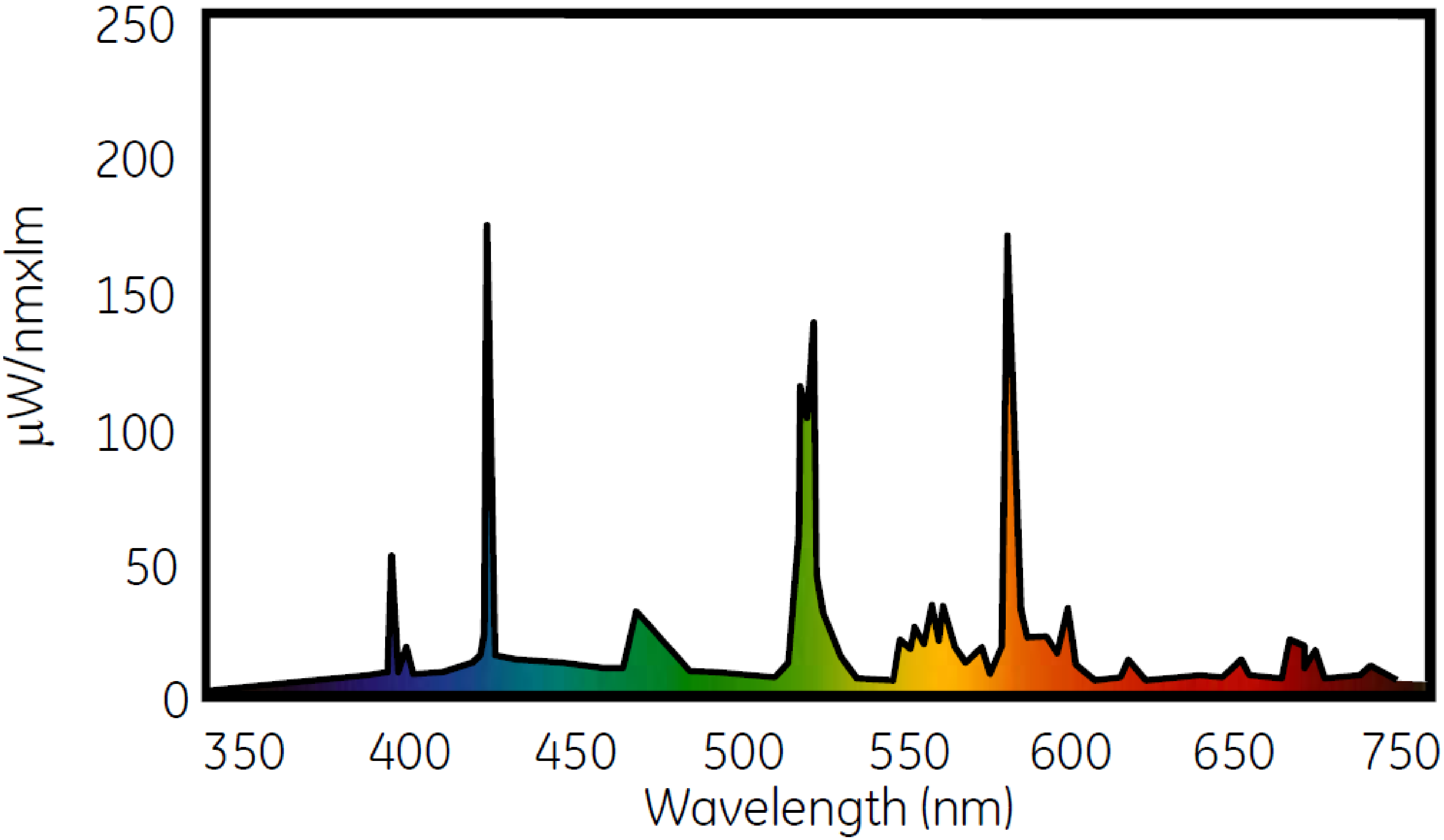


**Figure S1**. Spectral distribution on General Electric model T8 Ultramax F28T8-SPX41.

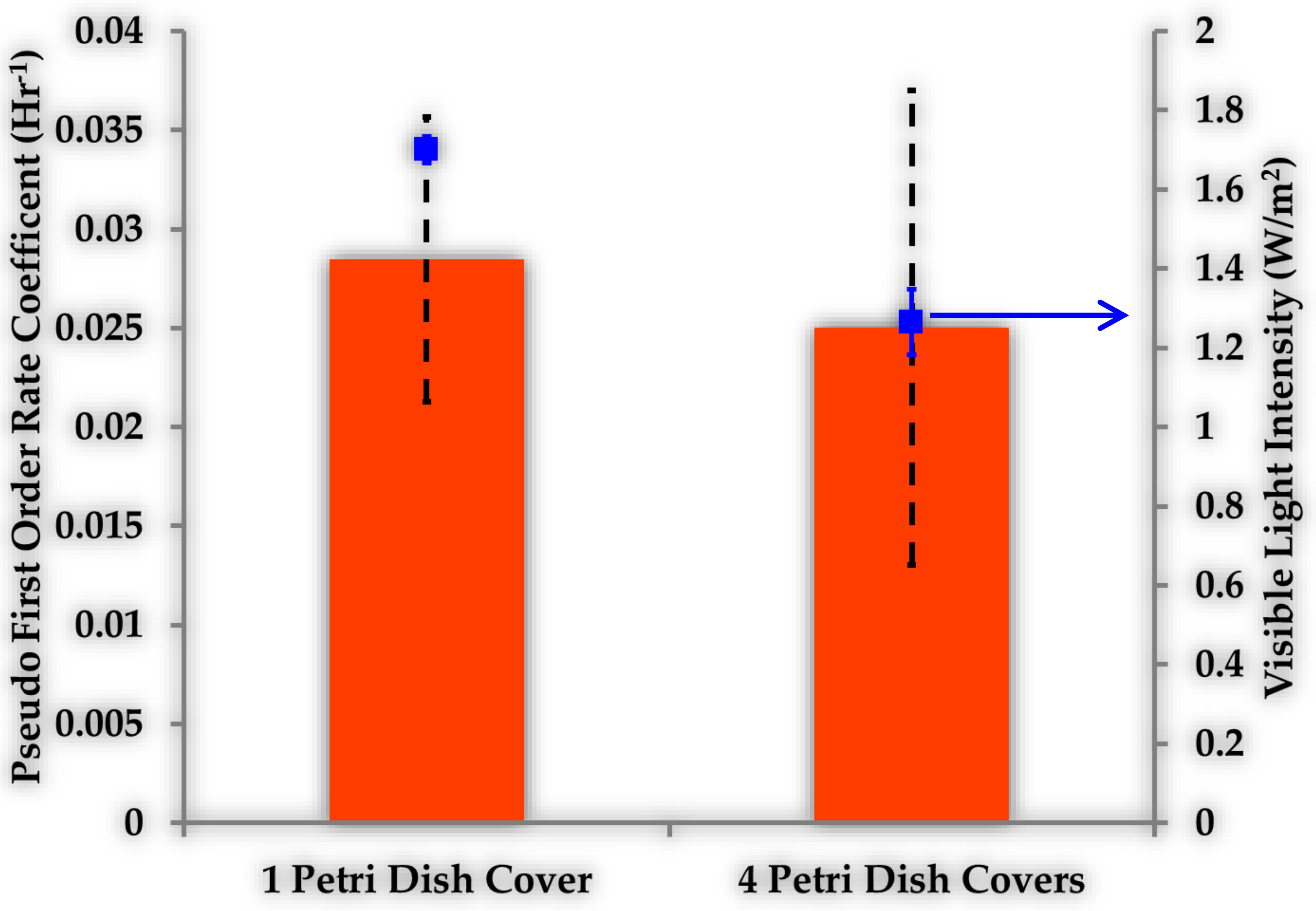


**Figure S2**. Effect of visible light intensity on pseudo first order rate coefficients for Mordant Orange degradation on anatase coating. No statistically significant decrease in rate coefficient is observed with 26% decrease in visible light intensity. N=4

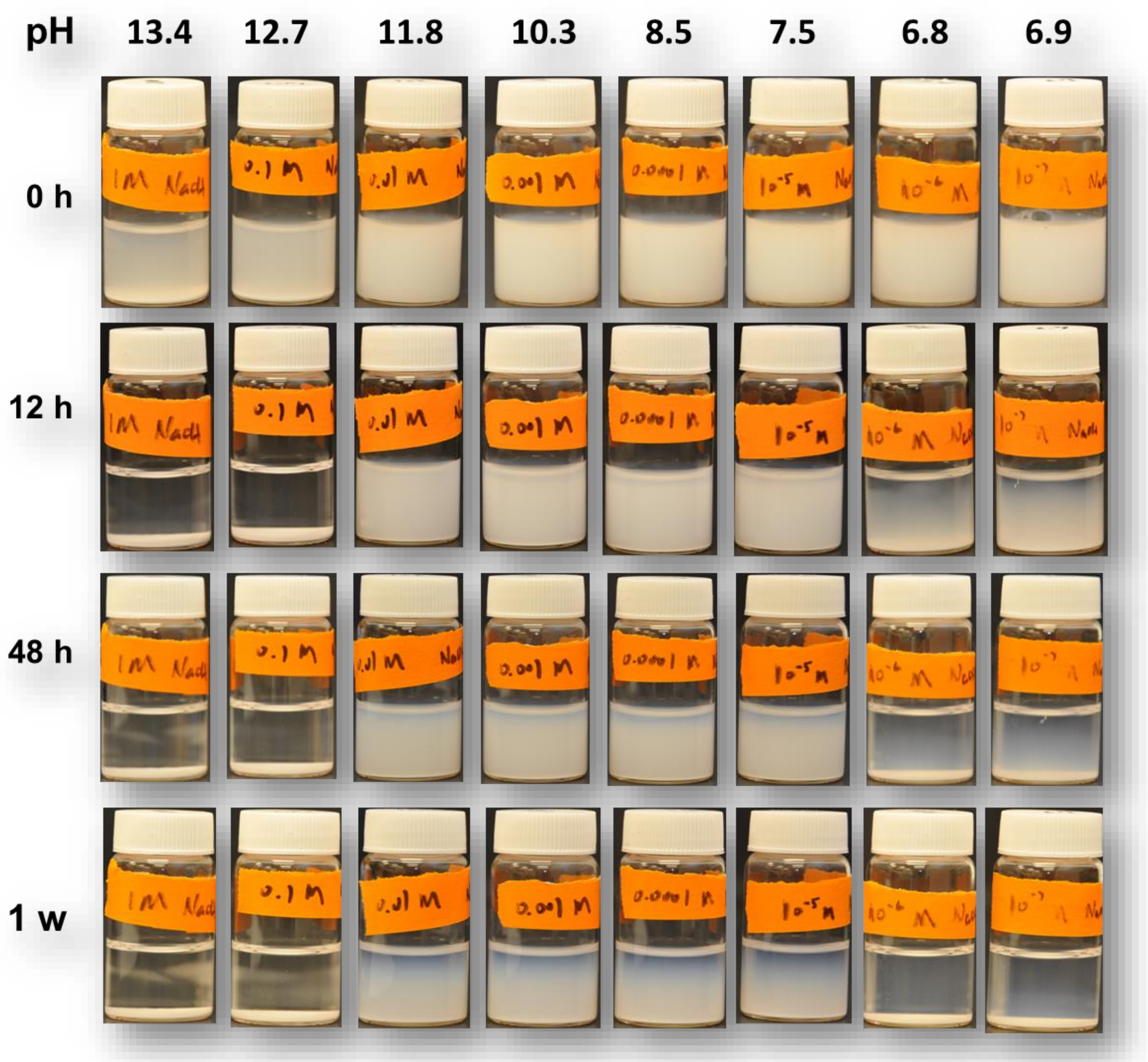


**Figure S3**. Temporal stability of $TiO_2$ suspension at different pH.

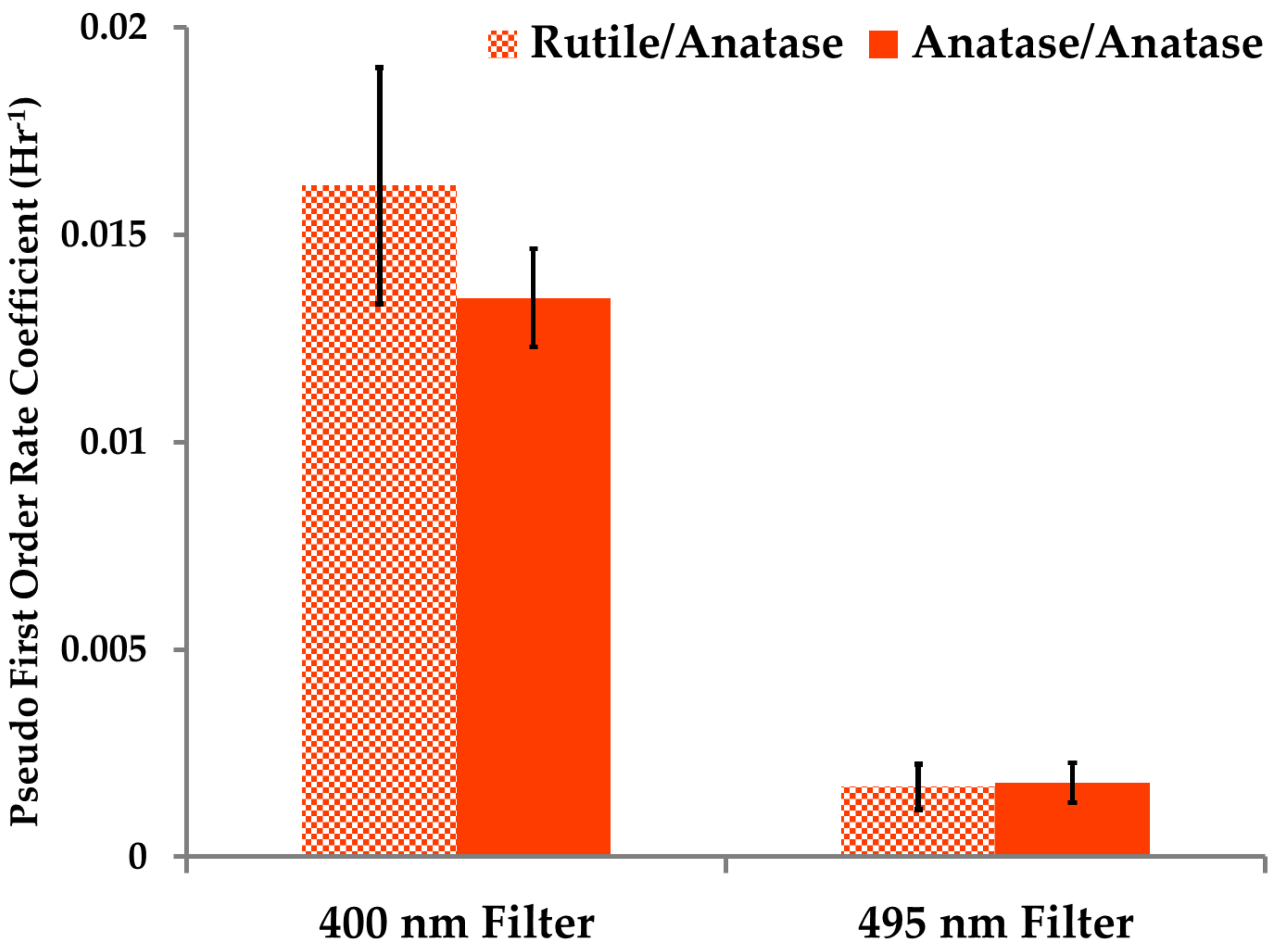


**Figure S4**. Effect of titanium dioxide crystal polymorph on pseudo first order rate coefficients for Mordant Orange degradation on anatase/anatase and rutile/anatase coatings. No statistically significant increase in rate coefficient is observed with replacement of anatase with rutile. N=10
